# Phytolith Radiocarbon Dating: A Review of Previous Studies in China and the Current State of the Debate

**DOI:** 10.3389/fpls.2019.01302

**Published:** 2019-10-16

**Authors:** Xinxin Zuo, Houyuan Lu

**Affiliations:** ^1^State Key Laboratory for Subtropical Mountain Ecology of the Ministry of Science and Technology and Fujian Province, Fujian Normal University, Fuzhou, China; ^2^School of Geographical Sciences, Fujian Normal University, Fuzhou, China; ^3^Key Laboratory of Cenozoic Geology and Environment, Institute of Geology and Geophysics, Chinese Academy of Sciences, Beijing, China; ^4^Center for Excellence in Tibetan Plateau Earth Science, Chinese Academy of Sciences, Beijing, China; ^5^College of Earth and Planetary Sciences, University of Chinese Academy of Sciences, Beijing, China

**Keywords:** older carbon, PhytOC, radiocarbon dating, phytolith age, phytolith

## Abstract

Phytolith radiocarbon dating can be traced back to the 1960s. However, its reliability has recently been called into question. Piperno summarized recent dating evidence, but most phytolith dating results from China were not included in the review because they are written in Chinese. Herein, we summarize and evaluate previous phytolith dating results from China. We also review recent debates on the nature and origin of phytolith-occluded carbon (abbreviated as PhytOC), as well as the older age of phytoliths retrieved from modern plants. We conclude that although PhytOC includes a small amount of old carbon absorbed from the soil, this carbon fraction has not always biased phytolith ages, indicating that in certain situations, phytoliths can be tried as an alternative dating tool in archaeological and paleoecological research when other datable materials are not available.

## Introduction

Phytoliths are noncrystalline SiO_2_ · nH_2_O that are deposited within the cells and cell walls in different parts of plants (Piperno, 2006). The morphology of a phytolith often resembles the shape of the cell in which it is formed and can be used in plant taxonomy. Phytoliths occlude a small amount of carbon during their deposition [phytolith-occluded carbon (PhytOC)] (Smith and Anderson, 2001; Parr and Sullivan, 2005). When a plant dies and decays, phytoliths and their occluded carbon can persist in the soil for a long time owing to the high resistance of phytoliths against decomposition. Phytolith analysis has been applied to environmental, anthropological, and geological research. Radiocarbon dating of phytoliths is a long-established technique that can be traced back to the 1960s (Wilding et al., 1967; Kelly et al., 1991; Piperno and Becker, 1996; Piperno and Jones, 2003). During the past decades, several researchers have attempted to date phytoliths, and some of them have achieved reasonable results. However, some of them have failed, because they found that phytolith carbon comes from multiple sources (either photosynthetic or soil carbon) (Reyerson et al., 2016). Moreover, the carbon in phytoliths that is taken up from the soil is variable and generally unknowable, which limits phytoliths carbon as a reliable dating material (Alexandre et al., 2015; Alexandre et al., 2016; Santos et al., 2018). Consequently, along with organic matter in pottery, phytoliths are considered as problematic samples for radiocarbon dating (Taylor and Bar-Yosef, 2014).

Recent debates in phytolith carbon dating research include the following topics: Is phytolith dating reliable? Is all phytolith carbon encapsulated *via* photosynthesis from atmospheric CO_2_ during plant growth, or is some absorbed from soil, which might distort phytolith dating? These questions are relatively new and were widely discussed recently (Hodson, 2018). Researchers have so far failed to reach an agreement on the reliability of phytolith carbon dating, largely because the scientific study of the nature, content, and status of PhytOC is still in its infancy.

In a review article, Piperno (2016a) summarized and evaluated almost all previous phytolith dating results of studies from different regions of the world. However, the results of several phytolith dating studies from China were not included, possibly because they are written in Chinese. Herein, we briefly review the history of phytolith carbon dating research. We then introduce and summarize the history of phytolith carbon dating research in China. Finally, we will discuss the main focus of current debate and the issues associated with phytolith carbon dating.

## A Brief History of Phytolith Carbon Dating Research

**Figure 1:**
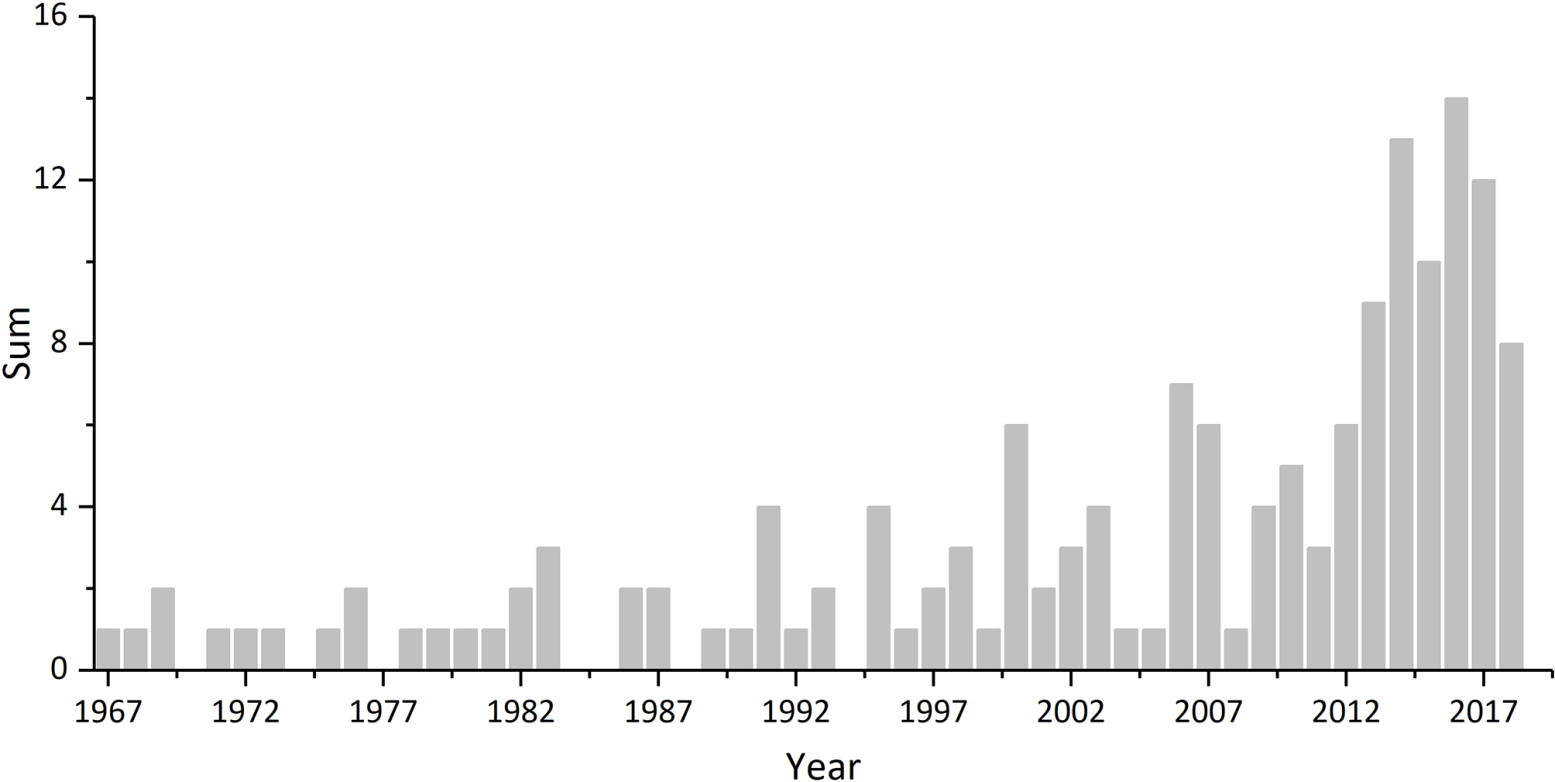
Number of articles that cited the study of Wilding *per* year after 1967. All references were collected from Google Scholar (https://scholar.google.com).

Jones and Beavers (1964) were the earliest researchers to discover that phytoliths can occlude carbon during their formation in plants (Wilding et al., 1967). The earliest attempt to date carbon in phytoliths was published in 1967 by Wilding (1967), who extracted approximately 75 g of phytoliths from 45 kg of a prairie surface soil horizon, isolated the occluded carbon, and obtained a date of 13,300 ± 450 a BP. Since Wilding’s pioneering research on phytolith carbon dating, three stages of phytolith carbon dating research can been identified according to the total annual citations of Wilding’ s 1967 article (**Figure 1**).

First is the early research period, from around 1970 to 1990. As shown in **Figure 1**, although Wilding’s phytolith dating results received some attention sporadically, only a few studies used phytolith dating to construct chronological sequences, mainly because of the time-consuming phytolith extraction process and the large sample size required for conventional radiocarbon dating.

Second is the revived period of research, from 1990 to 2010. The development of accelerator mass spectrometry (AMS) technology has enabled the measurement of very small samples containing trace amounts of carbon. Utilizing this technique, a much smaller amount of phytoliths would yield sufficient carbon for dating, greatly reducing the amount of phytolith extraction required. Mulholland and Prior (1993) summarized the process of AMS-based radiocarbon dating of phytoliths by presenting details of extracting and dating phytoliths. The initial application of phytolith carbon dating during this period was performed by Kelly et al. (1991). They applied phytolith carbon dating into three soil profiles from the northern Great Plains. The results showed that there may be some serious problems with dating phytoliths because two of the three soils they examined showed the phytoliths were younger at deeper horizons in the soil profile, contrary to expectations. Piperno and Stothert (2003) used phytolith carbon to date *Cucurbita* domestication through phytolith carbon-14 study during the early Holocene in Southwest Ecuador (Piperno and Becker, 1996; Piperno and Jones, 2003).

Third is the period of controversy in phytolith carbon dating research after 2010. Recent studies on phytolith dating of modern plants have argued that old carbon absorbed by plants from soils distorts the accuracy of phytolith carbon dating, with modern plants producing phytoliths radiocarbon dates up to several thousand years (Santos et al., 2010; Santos et al., 2012; Yin et al., 2014; Reyerson et al., 2016). Because the age of the phytoliths is overestimated compared with that of other dating materials, phytolith carbon is considered problematic for dating by several researchers (Taylor and Bar-Yosef, 2014; Santos et al., 2018). Other researchers argue that some reasonable phytolith dates have been measured from both modern plants and paleo-soils (Sullivan and Parr, 2013; Piperno, 2016a; Asscher et al., 2017; Zuo et al., 2017). Meanwhile, the soil phytolith ages extracted from different cultural layers of several archaeological sites have shown good consistency with their paired dating samples collected from the same depth (Asscher et al., 2017; Zuo et al., 2017) (**Table 1**).

**Table 1:** Researchers involved in phytolith carbon dating studies.

Authors	Institution	Dating materials	Processing method	References
L. P. Wilding	Department of Agronomy, Ohio State University	Well-drained Brunizem soil, Ohio	H_2_O_2_ + HCl (1N)	(Wilding, 1967)
E. Kelly	Department of Agronomy, Colorado State University	Prairie soil, Kansas and Nebraska	H_2_O_2_ + HCl (6N)	(Kelly et al., 1991)
D. Piperno	Smithsonian Tropical Research Institute, Balboa, Panama	Living plants, paleo-soil, Central America	HCl (1N) + H_2_SO_4_ or HNO_3_/KClO_3_	(Piperno and Stothert, 2003; Piperno, 2016a; Piperno, 2016b)
S. Mulholland	Duluth Archaeology Center, University of Minnesota	Soil	H_2_O_2_ + HCl (1N) + H_2_CrO_4_ (1N)	(Mulholland and Prior, 1993)
C. Prior	National Isotope Centre, GNS Science, Lower Hutt, New Zealand	Tephra, New Zealand	Not given in detail	(Santos et al., 2016)
G. Santos	Earth System Science, University of California, Irvine	Living plants; volcanoclastic soil, hydromorphic soil, ferralitic soil	H_2_O_2_ + HNO_3_ + HClO_4_ + HCl HNO_3_ + HClO_4_ + HCl	(Santos et al., 2010)
P. Reyerson	University of Wisconsin–La Crosse, United States	Living plants	HCl + H_2_SO_4_ + H_2_O_2_ + HNO_3_/KClO_3_; HNO_3_ + HClO_4_	(Reyerson et al., 2016)
U. Rieser	School of Geography, Environment and Earth Sciences, Victoria University of Wellington	Tephra, New Zealand	Rigorous oxidation, not given in detail	(Rieser et al., 2007)
J. Parr, L. Sullivan	Southern Cross GeoScience, Southern Cross University	Living plants, fallen leaves, Australian	HCl + H_2_O_2_ + HNO_3_	(Sullivan and Parr, 2013)
E. Boaretto, Y. Asscher	D-REAMS Radiocarbon Laboratory, Weizmann Institute of Science	Living plants, paleo-soil, cultural layers, Israel	HCl (1N)	(Asscher et al., 2017)
M. Madella	Department of Archaeology and Anthropology, IMF, Spanish National Research Council	Paleo-soil, cultural layers, Sudan	H_2_O_2_ + HCl (1N)	(Madella et al., 2014)
H. Lu	Institute of Geology and Geophysics, Chinese Academy of Sciences	Paleo-soil, cultural layers, China	H_2_O_2_ + HCl (1N) + HNO_3_/KClO_3_	(Zuo et al., 2017)
X. Zuo	School of Geographical Science, Fujian Normal University	Paleo-soil, cultural layers, China	H_2_O_2_ + HCl (1N) + HNO_3_/KClO_3_; H_2_O_2_ + HCl (1N)	(Zuo et al., 2018)
X. Wu, H. Jin, X. Yan	School of Archaeology and Museology, Peking University	Paleo-soil, paleo-soil, cultural layers, China, rice field	H_2_O_2_ + HCl (1N)	(Jin et al., 2014; Yan, 2013)
J. Yin, X. Yang	Institute of Geology, China Earthquake Administration	Living plants, paleo-loess, China	H_2_O_2_ + HCl (1N) + HNO_3_ + NaClO_2_	(Yang, 2013; Yin et al., 2014)

## Phytolith Carbon Dating Research in China

Phytolith research began in the late 1980s in China (Wang and Lu, 1989; Lu and Wang, 1990; Wang and Lu, 1992), which is over 150 years after the first report of phytoliths in living plants by Struve in 1835. The first report of phytolith carbon dating in a Chinese journal was published by Wang and Lu (1997), two pioneer phytolith researchers, in 1997 (Wang, 1998). They introduced the idea of radiocarbon dating of PhytOC to China, as summarized in the review of Mulholland and Prior (1993). Wang aimed to determine the chemical composition of phytoliths extracted from 16 species using an electron microprobe. Although the method used could not accurately measure the chemical composition of phytoliths, Wang was the first scholar in China who realized the importance of chemical aspects of phytoliths. However, both Wang and Lu did not actually date phytoliths.

It was only after 2010 that PhytOC and phytolith radiocarbon dating were studied again in China. To test the importance of carbon sequestration in phytoliths (Parr and Sullivan, 2005; Parr et al., 2009; Parr et al., 2010), we used the wet oxidation method to extract phytoliths from eight species of millet and showed a significant variation in PhytOC in different millet species (Zuo and Lü, 2011). Song et al. (2014; Song et al., 2017a) evaluated PhytOC and estimated the PhytOC accumulative rate in different ecosystems in China and even at the global scale. Zuo et al. (2014) then focused on soil phytoliths in the Chinese Loess Plateau, developing a wet oxidation method, modified from previous phytolith extraction processes, which can extract pure phytoliths from the soil (Piperno, 2006; Carter, 2009; Santos et al., 2010).

In 2013, Wu, an expert in archeometry from Peking University, cooperated with us in phytolith carbon dating by providing secure cultural layers rich in phytoliths. We then used the modified wet oxidation method to extract phytoliths, and the recovered phytoliths were sent to the Peking University Radiocarbon Laboratory for radiocarbon measurement. Wu also sent her students to our laboratory to learn how to extract pure phytoliths from soil. One of them, Jin, extracted phytoliths from the early cultural layers of Tianluoshan site. The results showed that the phytolith date (4,550 ± 35 a BP) was marginally older than their paired seeds age (4,400 ± 40 a BP). They speculated that the organic material with carboxyl groups that were not completely removed during the extraction processes might cause phytolith dates older than its paired seed date (Jin et al., 2014). Another student, Yan (2013), further compared different dating substances, such as charcoal, phytoliths, fatty acids, and total organic carbon, collected from the same depth of storage pits in Cishan site and paleo rice fields in Shanlonggang site. Among the five paired dating samples, two phytolith dates overlapped with their paired charcoal ages within ±2σ uncertainty; one was almost 5,000 years older than its paired charcoal age, and the remaining two were approximately 100 years older than the charcoal ages (**Table 2**). She concluded that the phytolith age is usually older than the charcoal age, while the fatty acid age was closer to the charcoal age, as it is relatively stable among all the dating substances (Yan, 2013).

**Table 2:** Phytolith radiocarbon dating results from China with uncertainty ±2σ.

Archaeological sites	Conventional age (BP)	2σ Calibration (Cal BP)	Reference
Shangshan	19,060 ± 60	23,065–22,825	Zuo et al., 2018
Shangshan	19,920 ± 70	24,115–23,830	
Hehuashan	10,800 ± 40	12,740–12,680	
Zhuangling	7,470 ± 30	8,370–8,200	
Guangtaoyuan	6,680 ± 30	7,590–7,505	
Miaoshan	7,720 ± 30	8,560–8,425	
Maanhe	5,310 ± 30	6,275–6,235	
Wuluoxipo	6,350 ± 30	7,506–7,417	Zuo et al., 2016
Tianluoshan	5,940 ± 30	6,805–6,674	
Tianluoshan	5,180 ± 30	5,990–5,906	
Xinglefang	5,110 ± 30	5,829–5,750	
Yuancun	5,310 ± 30	6,184–5,996	
Yingyang	5,760 ± 40	6,659–6,465	
Shangshan	8,280 ± 40	9,417–9,134	Zuo et al., 2017
Shangshan	7,280 ± 40	8,175–8,012	
Hehuashan	8,130 ± 40	9,121–8,992	
Hehuashan	8,040 ± 30	9,030–8,762	
Huxi	7,310 ± 40	8,186–8,021	
Huxi	7,180 ± 40	8,152–7,934	
Huxi	7,530 ± 30	8,406–8,221	
Huxi	7,680 ± 30	8,540–8,412	
Huxi	7,870 ± 40	8,953–8,553	
Tianluoshan	4,550 ± 35	5,190–5,052	Jin et al., 2014
Shanlonggang	2,370 ± 70	2,712–2,306	Yan, 2013
Shanlonggang	3,740 ± 40	4,197–4,232	
Cishan	10,890 ± 35	12,810–12,701	
Cishan	6,690 ± 40	7,622–7,478	
Cishan	7,285 ± 30	8,169–8,023	
Cishan	7,590 ± 35	8,433–8,346	
Cishan	8,725 ± 35	9,798–9,554	

Furthermore, Yin, an expert in quaternary geochronology from the Institute of Geology, Chinese Earthquake Administration, joined us in phytolith carbon dating. He and his colleague developed a new AMS graphite target preparation line in their ^14^C laboratory. They dated phytoliths extracted from paleo-loess with an OSL date of 71 ka. The results showed that the phytolith date (42,380 ± 180 a BP) was close to the background date of the graphite system (42,750 ± 190 a BP), suggesting that not only was soil PhytOC not contaminated by exogenous organic materials, but also very limited modern carbon was introduced during phytolith extraction, AMS graphite sample preparation, and radiocarbon measurement (Yang, 2013). They then combusted phytoliths extracted from modern rice and millet at different temperatures and the results showed that phytoliths combusted at lower temperatures (≤900°C) yielded more reasonable ages than at higher temperatures (≥1,100°C) (Yin et al., 2014). Given older phytolith ages at higher combustion temperatures, they speculated that there are probably two fractions of organic carbon in phytoliths, namely, labile and recalcitrant carbon.

As mentioned above, several Chinese research groups have shown great interest in phytolith carbon dating; however, only a few have provided images of phytoliths extracted from the soil to validate the efficiency of their extraction methods in completely eliminating all exogenous organic materials and other minerals. In this regard, we used our modified oxidation method to extract phytoliths from the cultural layers of several archaeological sites in China. Before sending the phytolith samples to Beta Analytic for radiocarbon measurement, we used scanning electron microscopy, energy-dispersive X-ray spectroscopy, and X-ray refraction to check the purity of phytoliths. The preliminary results showed that most of the phytolith ages were generally consistent with that of other dating materials collected from the same depth as phytolith samples, except for one outlier (Zuo et al., 2016). We attributed the inconsistency to the postdepositional processes of soil phytoliths. This suggests that each step of phytolith dating, including sampling, extracting, and measurement, should be carefully carried out to ensure that phytolith carbon dating is based on a secure archaeological context (without postdepositional processes) and appropriate chemical preparation (without exogenous organic materials). Our results showed that, for these sites, phytolith ages were consistent with those of other dated materials at the same level or context, suggesting that phytolith radiocarbon dating can be reliable and accurate at some sites (Zuo et al., 2017).

The reliability of phytolith dating will be discussed with respect to the following three aspects: 1) Is old carbon from the soil occluded into the phytoliths? 2) If so, how much will the old carbon skew the phytolith age determination? 3) Do the different methods (both for phytolith extraction and radiocarbon measurement) affect the phytolith dating results?

## The Nature and Source of Phytoc: Older Carbon or Photosynthetic Carbon?

Although there has been considerable discussion, researchers began to pay attention to the nature of PhytOC in the early stage of phytolith carbon dating research in the 1960s. Infrared spectral data of phytoliths suggested that PhytOC is composed of a variety of cell-derived substances, such as humic acid, amino acids, and amines (Wilding et al., 1967). The significantly depleted δ^13^C in phytoliths relative to that in the host plant tissue indicated that PhytOC might include lipids and lignin, which might have a depleted carbon isotope (Kelly et al., 1991). Smith and Anderson (2001) also found lipids in phytoliths, but no lignin. Masion et al. (2017) detected several carbohydrate components in phytoliths, such as sugars, adenosine triphosphate, and sodium pyrogluconate, using a new technique of dynamic nuclear polarization nuclear magnetic resonance. Raman spectrum analysis of single dumbbell phytoliths from sorghum also revealed that phytoliths contain carbohydrates, lipids, and other organic substances (Gallagher et al., 2015). Although there are differences in the understanding of the nature of PhytOC, previous studies assumed organic matter from plant tissue is the only source of PhytOC.

Santos, an expert in isotopic analysis, was the first to question the reliability of phytolith carbon dating. Initially, Santos et al. (2010) performed radiocarbon AMS measurement of carbon occluded in phytoliths from living plants and unexpectedly obtained dates that were several thousand years old. They suggested that there are some possible sources of carbon contamination, which needed further investigation (Santos et al., 2010). In 2012, they further suggested that soil-derived carbon (older carbon) absorbed by plant roots is a possible reason for the old phytolith ages obtained for living plants (Santos et al., 2012), although they lacked direct evidence showing that phytoliths can occlude older carbon from the soil. If older carbon is occluded in phytoliths, not only is the use of phytolith carbon for dating called into question, but it also reduces the importance of PhytOC in global carbon sequestration (Santos and Alexandre, 2017), and phytolith carbon sequestration might not be as significant as that reported by Song et al. (2016). While the contribution of old soil carbon to PhytOC was debated by several researchers interested in PhytOC (Piperno, 2016b; Santos et al., 2016; Santos and Alexandre, 2017; Song et al., 2017b; Zuo et al., 2017; Santos et al., 2018), Santos and her group were seeking direct evidence of soil-derived C in phytoliths. Using the comparative isotopic analysis of PhytOC, host tissues, atmospheric CO_2_, and soil organic matter, they found that PhytOC is partially obtained from soil carbon (Reyerson et al., 2016).

It is now clear that small amounts of soil carbon are occluded in phytoliths, as well as in plant tissues, as some hydroponic experiments have indicated that plants can absorb a small amount of sucrose or glucose from the source medium (Wu et al., 2015; Zhang and He, 2015; Chen et al., 2016). Because it is not possible to estimate the percentage of PhytOC that is of soil origin and the age of the soil carbon occluded by phytoliths is unknown, Santos et al. (2018) suggest that radiocarbon dating of phytoliths is highly problematic and not trustworthy.

## Contribution of Older Soil Carbon to Phytolith Ages

With further understanding of the nature of phytoliths and PhytOC, we now realize that although most of the PhytOC is from atmospheric CO_2_ fixed by photosynthesis, a small amount of carbon is not photosynthetic, likely derived from soil organic carbon. Because plants absorb old soil carbon through the roots, this carbon should be homogenously distributed in different tissues (Gallagher et al., 2015), and the roots, stems, leaves, and other parts will contain old carbon from the soil. If the phytolith ages are skewed by older carbon from the soil, one would expect the same effect when dating plant tissue, but this is clearly not the case, because plant debris is one of the best dating materials in sediment. Santos et al. (2018) noted that compared to PhytOC ^14^C results, plant-C ^14^C results were not biased by old soil carbon, suggesting the asymmetric ^14^C effects of soil carbon contribution to plant debris and PhytOC. They speculated that there must be some unknown processes that allow most of the soil carbon absorbed by the roots to accumulate in phytoliths (Alexandre et al., 2016; Santos et al., 2018). However, due to the limited knowledge about the relocation of soil carbon in plants, further studies are needed to investigate whether asymmetric relocation of soil carbon exists in plants. However, Hodson (2012; 2015)has stated that no mechanism can explain why soil carbon preferentially accumulates in phytoliths, while large amounts of photosynthetic carbon in plant tissues are excluded during the deposition of silica.

It is unreasonable to attribute all questionable phytolith-dating results to distortion by soil carbon. Other possible factors influencing the process of sampling, phytolith extraction, and radiocarbon measurement cannot be ignored when evaluating phytolith ages. Studies on contamination effects on ^14^C dating showed that the introduction of 1% dead carbon can only result in an increase in the age by approximately 80 years (Taylor and Bar-Yosef, 2014). With the isotopic-labeled analysis of the silicon-rich hydroponic solution of grass, it was revealed that soil-derived carbon in phytoliths might constitute 0.15% of the PhytOC (Alexandre et al., 2016). Even though the actual percentage is likely considerably higher in natural soil conditions, such a small amount of older carbon will not yield phytolith ages thousands of years older than expected if assuming a 1.5% soil carbon contribution to PhytOC (10 times higher than under hydroponic conditions).

## Influence of Different Extraction and Radiocarbon Measurement Methods on Phytolith Carbon Dating

The wet oxidation method is the main phytolith extraction method in phytolith carbon dating research, and the difference among different extraction methods is mainly in the oxidation stage before heavy-liquid flotation of phytoliths. One method uses H_2_SO_4_ + H_2_O_2_, known as rapid oxidation or over oxidation, and the other uses HNO_3_ or HNO_3_ + KClO_3_. Researchers who used the latter method suggested that the oxidation process should remove as much exogenous organic matter as possible; however, rapid oxidation is so harsh that it not only can remove the exogenous organic matter, but also might change the nature of phytoliths, thus skewing the phytoliths ages (Sullivan and Parr, 2013; Song et al., 2016; Zuo et al., 2016). Whether the rapid oxidation method will change the nature of PhytOC remains unclear, but the PhytOC content will decrease significantly after rapid oxidation (**Table 3**) (Santos et al., 2010; Zuo and Lü, 2011; Santos et al., 2012; Corbineau et al., 2013), indicating that the carbon occluded in cavities of phytoliths is likely to be removed and that the integrity of PhytOC is destroyed (Sullivan and Parr, 2013; Parr and Sullivan, 2014).

**Table 3:** Several species of PhytOC content in phytoliths processed by different oxidation methods.

Species	The oxidation methods	PhytOC of phytoliths (%)	Reference
Reed	Less harsh	0.66–2.44	(Li et al., 2013a)
Rice	Less harsh	1.4–3.4	(Li et al., 2013b)
Bamboo	Less harsh	1.60–4.02	(Parr et al., 2010)
Wheat	Less harsh	1.29–12.91	(Parr and Sullivan, 2011)
Wheat	Less harsh	1.65	(Hodson et al., 2008)
Sugarcane	Less harsh	3.88–19.26	(Parr et al., 2009)
Sandy grassland	Less harsh	0.57–1.55	(Ru et al., 2018)
Millet	More harsh	0.88–4.88	(Zuo and Lü, 2011)
Festuca	More harsh	0.07–0.15	(Carter, 2009)
*Sorghum*, wheat	More harsh	0.002–0.24	(Reyerson et al., 2016)

The overoxidation method is so strong that it might cause phytolith ages older than the expected ages because of changes in the nature and structure of PhytOC, while the underoxidation method and incomplete removal of organic material could cause older phytolith ages (Zuo et al., 2018). We compared the influence of two different phytolith extraction methods on radiocarbon dating of phytoliths. The results showed that phytolith ages acquired using the conventional extraction method that does not exclude all exogenous organic materials were substantially older than those obtained using improved extraction methods.

Nondestructive phytolith extraction methods to extract phytoliths without using a strong acid not only can yield pure phytoliths, but also can maintain the integrity of PhytOC. Asscher et al. (2017) only used HCl (1N) to exclude calcium carbonate in the phytolith extraction process. Before acid treatment, they used a heavy liquid (2.4 and 1.6 g/ml) to remove quartz, calcite, and carbonized organic matter. There was no heating in any step of the phytolith extraction process. The results showed that several phytolith ages were consistent with the age of carbonized seeds within the ±1σ correction interval at the same level; the others have slightly older ages (Asscher et al., 2017). These phytolith dates were challenged by Santos et al. because they were obtained from phytoliths whose purity was not assessed by scanning electron microscopy (SEM) and energy-dispersive X-ray spectroscopy (EDX) (Santos et al., 2018). The non-heating method used by Asscher et al. (2017) is not likely to produce a pure phytolith extract and thus the remaining exogenous organic matter might cause phytolith ages older than the paired seeds ages. This method was also used to extract phytoliths in the analysis of DNA in phytoliths, in order to avoid the influence of strong acids and high temperatures on DNA information that might be preserved in phytoliths (Elbaum et al., 2009).

## Possible Reasons for Older Phytolith Ages in Soil Profiles and Living Plants

After carefully reviewing previous phytolith dating reports and other studies where older carbon may have biased PhytOC dates (Kelly et al., 1991; Piperno and Becker, 1996; Kelly et al., 1998; Krull et al., 2003; Piperno and Jones, 2003; Piperno and Stothert, 2003; Mcmichael et al., 2012; Sullivan and Parr, 2013; Madella et al., 2014), we speculate that older phytolith ages in soil profiles could be explained by the following two aspects. First, if the extracted phytoliths are pure after checking with SEM and EDX, then the postdepositional processes of phytoliths should be considered. Second, if the phytolith dating results are older than expected (even ten thousand years older), one should repeat the experiment and revaluate if the protocols used could exclude all carbonate and other minerals from the samples. Incompletely excluding sources of dead carbon can lead to phytolith ages hundreds to thousands of years older than expected. The introduction of 5% old carbon would make the dating sample (true age is 10,000 a BP) approximately 400 years older, and adding 50% very old carbon would make the age only about 5,000 years older. Thus, if no more than 1% PhytOC is taken up from the soil, one would not expect phytolith dates to differ from expected ages by thousands of years.

**Figure 2:**
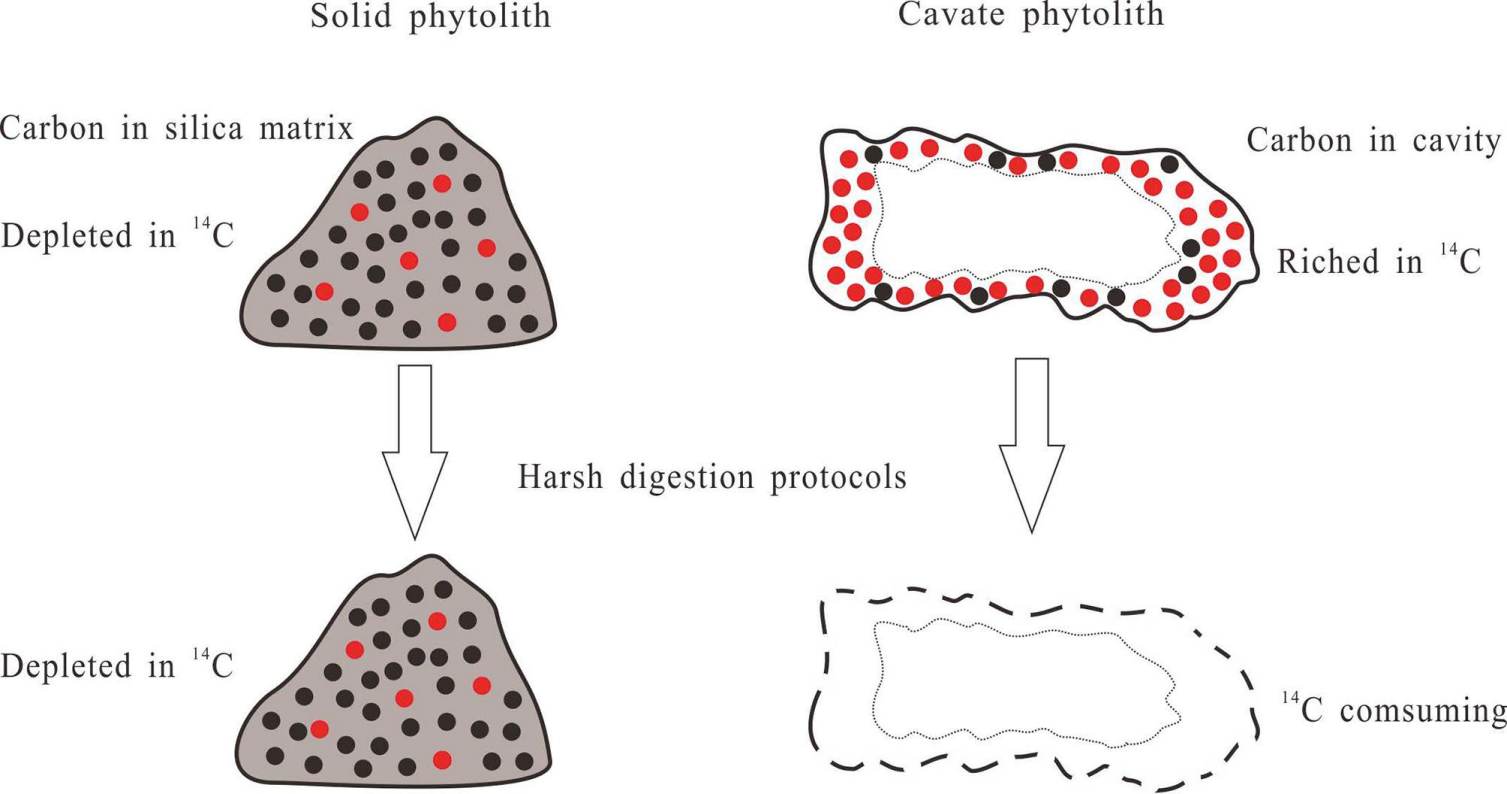
Influence of harsh digestion protocols on phytolith carbon from different kinds of phytoliths. The red and black dots represent ^14^C and ^13^C, respectively.

The unexpectedly older ages dated for extracted phytoliths from modern plants (Reyerson et al., 2016) may be caused by phytolith extraction procedures such as overaggressive digestion protocols that alter the structure, nature, and yield of PhytOC (Sullivan and Parr, 2013; Parr and Sullivan, 2014). Recently, some detection techniques, such as Raman spectroscopy and nanoscale secondary ion mass spectrometry, have been used to determine the location, distribution, and chemical structure of PhytOC (Alexandre et al., 2015; Gallagher et al., 2015), showing a continuous but nonhomogeneous distribution. The amount and nature of PhytOC might vary considerably depending on phytolith morphology and different allocations within phytoliths. Using harsh protocols to extract phytoliths from modern plants (Santos et al., 2010; Santos et al., 2012; Corbineau et al., 2013; Yin et al., 2014; Reyerson et al., 2016) might damage the integrity of PhytOC. Moreover, the carbon in cavate or surface of phytoliths might be consumed by the harsh digestion protocols, which would lead to underestimation of the total amount of PhytOC (Parr and Sullivan, 2014). The consumed carbon might be isotopically rich in ^14^C; however, the residual carbon fraction might be highly depleted in ^14^C (**Figure 2**). Given that the lipids within phytoliths are depleted in ^13^C (Smith and Anderson, 2001), this is also probably true for ^14^C (Hodson, 2016).

## Reconciling or Rebutting?

As mentioned above, it is difficult for researchers to reconcile on the reliability of phytolith carbon dating. The focus of discussion is mainly on the nature of PhytOC, the actual contribution of soil carbon to phytolith dates, and influences of different extraction and measurement processes on phytolith dates. Santos et al. based their older carbon theory on the following four aspects: 1) the age of phytoliths from modern living plants are decades to thousands of years older than their sampling time (Santos et al., 2010; Santos et al., 2012); 2) over 200 comparative isotopic measurements of PhytOC and isotopic-labeled experiment provide evidence of soil carbon in PhytOC (Reyerson et al., 2016); 3) although soil carbon can be absorbed by the roots, it does not skew plant-C ^14^C results, but only the PhytOC ^14^C results; and 4) no matter how soil phytolith dates match their expected ages, they are all questionable due to the variability of soil carbon contribution to PhytOC (Santos et al., 2018).

As discussed in the beginning of the review, not all phytolith dating results are older than the expected results. Several phytoliths extracted from modern plants, dated by Piperno (2016a) and Sullivan and Parr (2013), have either returned postbomb ^14^C ages or are very close to the modern dates. Most of the older modern phytoliths were dated by Santos et al. (2010). Phytolith dates from modern plants processed with the harsh techniques (Santos et al., 2012; Reyerson et al., 2016) are often considerably older than on plants processed with less harsh methods (Piperno, 2016a; Asscher et al., 2017; Zuo et al., 2017). Moreover, the harsh techniques typically leave much less carbon for dating than less harsh methods, partially due to leakage and the dual source of carbon—one labile and the other resistant (Hodson, 2019). Although researchers have stated that they have carefully dated PhytOC, Santos et al. (2012) might have only dated the carbon in lumen phytoliths, while Piperno et al. (2016a) might have dated not only the carbon in lumen phytoliths but also a part of carbon in cell wall phytoliths. A high amount of carbon processed by less harsh methods might preserve the integrity of PhytOC, but a less amount of carbon processed by harsher methods should not be preferred for dating.

Another key point that must be considered is that phytoliths differ in several aspects from other datable materials such as charcoal and seeds. Dating phytoliths and charcoal from the same stratigraphic/sedimentary level does not mean that they should have exactly the same dates, since phytolith age is the average age of all phytoliths in that level, whereas macro-plant/charcoal dates from a single sample represent a single moment in time. It is unreasonable to expect that a piece of charcoal or seed deposited at a single moment can completely fall within the age of a collection of phytoliths (Piperno, 2016a). A difference of hundreds of years between the dating results of soil phytoliths and other datable materials when sampling a thick soil layer of 5 to 10 cm is generally acceptable and reasonable (Zuo et al., 2016). Considering the depositional processes of phytoliths in soil, PhytOC should not be used for answering high-resolution chronological questions. However, it can be tried as an alternative dating method when other datable materials are absent.

## Conclusions and Remarks

As an unconventional ^14^C dating material, phytoliths have been widely used during the past half century. Radiocarbon dating PhytOC has played an important role in constructing the chronological sequence of some key scientific issues, such as when pumpkin and rice domestication began (Piperno and Stothert, 2003; Zuo et al., 2017), but at the same time, the technique has also been criticized (Santos et al., 2016; Santos and Alexandre, 2017; Santos et al., 2018). A review of the phytolith dating literature revealed that not all phytolith dating results are inconsistent with expected ages. The poor results cannot be entirely attributed to the influence of older carbon absorbed from the soil, because most of the PhytOC is obtained from the atmospheric CO_2_ synthesized by photosynthesis. Phytolith ages thousands of years older than expected are probably due to impure phytolith extracts not completely cleaned of extraneous carbon rather than phytolith occluded carbon obtained from the soil.

Compared with other conventional dating materials, research on the mechanisms, methods, and results of phytolith dating is limited. There are considerable empirical data showing that at many sites, PhytOC dating provides reasonable dates. However, concerns about extract purity, as well as the variable nature of the PhytOC carbon pool, suggest that the reliability of phytolith dates is questionable in many cases. Whether different phytolith extraction methods will inevitably lead to differences in the dating results remains an open question. Whether the difference in the PhytOC content obtained using the rapid oxidation method and the conventional oxidation methods is due to PhytOC being destroyed or the organic matter in plants being incompletely removed is important for evaluating the phytolith dating results and key to reconcile the conﬂicting opinions. Phytolith researchers working with PhytOC urgently need to agree on a standardized extraction procedure that produces a phytolith extract verified by SEM and EDX to be free of extraneous carbon while using the least harsh chemicals possible. We expect that more data on phytolith dating in other regions and laboratories will be published in the future and will further clarify issues relating to ^14^C dating and will allow the expansion of the application of phytolith dating to the construction of chronological sequences.

## Author Contributions

XZ designed and wrote the manuscript. HL contributed to discussion and approved the final manuscript.

## Funding

This work is jointly supported by the National Natural Science Foundation of China (41771241 and 41830322) and the Innovation Research Team Fund of Fujian Normal University (IRTL1705).

## Conflict of Interest

The authors declare that the research was conducted in the absence of any commercial or financial relationships that could be construed as a potential conflict of interest.
